# Dynamic Recrystallization and Its Effect on Superior Plasticity of Cold-Rolled Bioabsorbable Zinc-Copper Alloys

**DOI:** 10.3390/ma14133483

**Published:** 2021-06-23

**Authors:** Anna Jarzębska, Łukasz Maj, Magdalena Bieda, Robert Chulist, Daniel Wojtas, Maria Wątroba, Karol Janus, Łukasz Rogal, Krzysztof Sztwiertnia

**Affiliations:** 1 Institute of Metallurgy and Materials Science, Polish Academy of Sciences, 30-059 Krakow, Poland; l.maj@imim.pl (Ł.M.); m.bieda@imim.pl (M.B.); r.chulist@imim.pl (R.C.); Daniel.Wojtas@fis.agh.edu.pl (D.W.); k.janus@imim.pl (K.J.); l.rogal@imim.pl (Ł.R.); k.sztwiertnia@imim.pl (K.S.); 2 Faculty of Physics and Applied Computer Science, AGH University of Science and Technology, 30-059 Krakow, Poland; 3 Faculty of Metals Engineering and Industrial Computer Science, AGH University of Science and Technology, 30-059 Krakow, Poland; mwatroba@agh.edu.pl

**Keywords:** bioabsorbable zinc, microstructure, superplasticity, dynamic recrystallization

## Abstract

High plasticity of bioabsorbable stents, either cardiac or ureteral, is of great importance in terms of implants’ fabrication and positioning. Zn-Cu constitutes a promising group of materials in terms of feasible deformation since the superplastic effect has been observed in them, yet its origin remains poorly understood. Therefore, it is crucial to inspect the microstructural evolution of processed material to gain an insight into the mechanisms leading to such an extraordinary property. Within the present study, cold-rolled Zn-Cu alloys, i.e., Zn with addition of 1 wt.% and 5 wt.% of Cu, have been extensively investigated using scanning electron microscopy as well as transmission electron microscopy, so as to find out the possible explanation of superior plasticity of the Zn-Cu alloys. It has been stated that the continuous dynamic recrystallization has a tremendous impact on superior plasticity reported for Zn-1Cu alloy processed by rolling to 90% of reduction rate. The effect might be supported by static recrystallization, provoking grain growth and thereby yielding non-homogeneous microstructures. Such heterogeneous microstructure enables better formability since it increases the mean free path for dislocation movement.

## 1. Introduction

Zn-based biodegradable alloys have enjoyed a soaring popularity in research over the last couple of years [1,2]. This is mostly because of their compatibility with blood and tissues as well as corrosion rates that could easily go up against those demonstrated by Mg- and Fe-based biodegradable materials [3]. However, in the as-cast state, Zn and its alloys exhibit abnormally low mechanical properties, and therefore their wide-scale use becomes obstructed. Alloying combined with grain refinement, obtained by e.g., severe plastic deformation (SPD) technologies, has turned into an effective solution eliminating the aforementioned drawbacks [1]. A host of alloying elements, including Mg, Ca, Sr, or Li, have been introduced, yielding a wide range of biodegradable metallic materials for medical applications [4]. However, a careful selection of alloying elements is crucial as some of them (e.g., Ag or Al) tend to exceptionally strengthen pure Zn, although they may pose a threat to a human body. In fact, Ag might be toxic at high concentrations, while Al is debated to have a negative impact on the nervous system [5,6]. Therefore, Cu was proposed as the alloying element.

Copper is an essential microelement of rich history in medical use as a therapeutic or antibacterial agent. It is required for survival and serves as a cofactor for a variety of life-sustaining proteins and metalloenzymes [7,8]. It has been reported that various Cu-bearing Zn alloys manifest good mechanical properties as well as superplasticity [9,10,11], a feature of vast importance in terms of implants’ fabrication and positioning. Not only bone implants, but also wound closure devices and stents could make use of materials able to harmlessly dissolve in a human body. In fact, biodegradable metals have been mostly proposed as candidates for cardiovascular stents since their usage significantly increases year by year and a long-time service of a stent in clogged artery is not necessary [2]. On the other side, ureteral stents are rarely addressed while researching biodegradable materials [12]. Considering their implantation site, Zn-Cu alloys may be an appropriate choice for the production of ureteral stents due to their antibacterial properties. It is also worth mentioning that in comparison with pure Zn, Zn-Cu alloys are characterized by slightly higher corrosion rates, enabling a material to degrade faster, which is also favorable in terms of ureteral stents [13]. A huge advantage of the Zn-Cu system is its high ductility, needed while a stent is inserted into the ureter.

Thus, within the present study, cold-rolled Zn alloys with the addition of 1 wt.% and 5 wt.% Cu have been studied in detail. Although biodegradable metals are typically investigated with regard to their corrosion and biological behavior [14,15,16,17], it is crucial to control the microstructure of a material as it reflects on a host of properties. Moreover, investigation of microstructure evolution during plastic deformation can shed some light on the understanding of deformation mechanisms of the processed materials, yet such an approach has been barely studied in terms of Zn-Cu alloys. Therefore, careful microstructure examination of processed Zn-Cu alloys was carried out using state-of-the-art techniques such as electron backscatter diffraction (EBSD) and transmission electron microscopy (TEM). Finally, the mechanical properties of the obtained materials were assessed by a set of static tensile tests. The main goal was to reveal the origin of superior plasticity observed in Cu-bearing Zn alloys, as it has not been completely understood so far. 

## 2. Materials and Methods

Zn-1Cu and Zn-5Cu (in wt.%) alloys, used in the presented experiments, were produced by the gravity casting method. Zinc of 99.99 wt.% purity and CuZn40 brass were heated up to 650 °C and melted in a chamotte crucible inside the Nabertherm N20/14 resistance furnace (Lilienthal, Germany) under argon atmosphere. Additionally, the melt was coated with borax in order to avoid oxidation. Next, the Zn-1Cu and Zn-5Cu materials were cast into the steel molds. As a result, cylindrical ingots with a diameter of 40 mm and a height of 170 mm were obtained. Subsequently, the ingots were hot-extruded at 280 °C with the reduction R = 16, yielding a rod with a diameter of 10 mm. Finally, the hot-extruded materials were subjected to multi-pass cold-rolling up to 90% of reduction at room temperature. In order to characterize the microstructural evolution, two additional deformation stages were examined, namely 50% and 75% of reduction. The reduction ratio of 3% for each rolling pass was executed, meaning 10 passes, 15 passes and 18 passes corresponding to the reduction rate of 50%, 75% and 90%, respectively. 

The microstructure characterization of the cold-rolled Zn-1Cu and Zn-5Cu alloys was done by means of orientation imaging microscopy (OIM) performed with the help of the EBSD method, using a FEI Quanta 3D 200i FEG-SEM microscope (Eindhoven, The Netherlands) equipped with an EDAX OIM TSL EBSD system ver. 7.0. (Berwyn, IL, USA). The analysis was done on the normal cross-section to the rolling direction (RD). The map of the size of 128 × 128 µm was collected with a step size of 100 nm. Additionally, for samples after the tensile test, data collection was performed on the rolling plane, where the orientation map with the size of 100 × 100 µm and a step size of 60 nm was gathered. For the sake of better visibility, the presented orientation maps in the results section were cropped to 60 × 60 µm; however, all of the acquired data was considered in the calculations (except the points described below). The orientation maps were analyzed using the TSL OIM ver. 7.0 computer software (Berwyn, IL, USA). The grain was defined as a set of at least five measurement points, characterized by the same orientation and separated from a neighboring grain by a high-angle grain boundary with the misorientation angle exceeding 15°. Additionally, the measurement points with a low confidence index (CI < 0.1) were removed from the calculations. Based on the collected data, grain size, grain boundary character as well as sub-grain characteristic, i.e., grain orientation spread (GOS), were analyzed. The GOS parameter describes the level of local misorientation within a particular grain, indicating distortions in the crystal lattice caused mostly by dislocations e.g., in the form of sub-grains. The higher the value of GOS, the more deformed microstructure has been obtained. On the contrary, a low GOS value represents recrystallized grains. The preparation of the metallographic cross-sections for the SEM/EBSD studies relied on the standard procedure, covering grinding with abrasive papers, ranging from 100 up to 7000 grit, followed by polishing with 1 µm and ¼ µm diamond suspension. The final step of samples’ preparation for SEM/EBSD measurements differed depending on the alloy composition. In the case of Zn-1Cu alloy, electropolishing executed by applying the Struers Lectro-Pol machine (Copenhagen, Denmark) with the C1 Struers electrolyte at 25 V for 15 s was performed. For the Zn-5Cu alloy, as the final step, the low-angle Ar^+^ ion polishing for 20 min and 3.5 kV, using a Hitachi IM4000Plus Ion Milling System (Tokyo, Japan), was performed to improve the EBSD pattern quality and remove the deformed layer after mechanical preparation.

Microstructure characterization in the nanoscale was executed with the help of FEI Tecnai G2 SuperTWIN FEG transmission electron microscope (Eindhoven, The Netherlands) operated at 200 kV. It is equipped with a SIS MegaView III CCD camera, a Fischione detector and an EDAX energy dispersive X-ray spectrometer (EDS) for the acquisition of microstructure images in the bright field (TEM/BF)/dark field (TEM/DF) modes, together with electron diffraction patterns, STEM/HAADF microphotographs and X-ray spectra for chemical microanalysis. The phase analysis was carried out based on the acquired selected area electron diffraction (SAED) patterns, using CSpot computer software. The samples for TEM inspections were prepared by means of the electropolishing method with the use of a Struers TenuPol-5 machine (Copenhagen, Denmark) with the electrolyte cooled down to −20 °C (5% of perchloric acid and 95% ethanol) operated at 30 V. In addition, a thin lamella was also cut out from the sample after the tensile test with the use of the focused ion beam (FIB) technique, carried out on a ThermoFisher Scios 2 Dual Beam microscope (Eindhoven, The Netherlands), equipped with an EasyLift^TM^ nanomanipulator.

**Figure 1:**
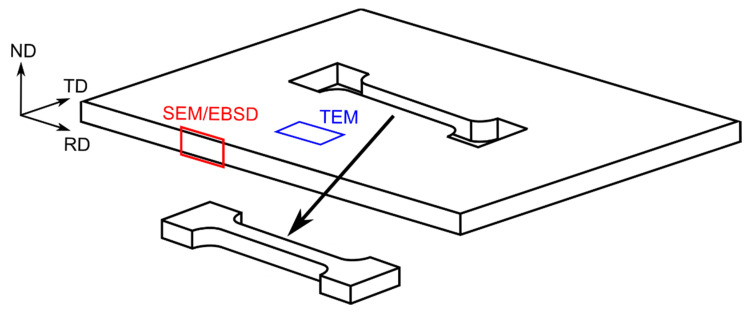
Scheme of cutting samples from Zn-Cu sheets for uniaxial tensile test and depicted cross-section of EBSD and TEM investigations.

Mechanical properties were determined by uniaxial static tensile tests. The tests were performed by using an Instron 6025 machine (Norwood, MA, USA) at a room temperature with a constant strain rate of 10^−3^ 1/s. For each cold-rolling parameter, three different samples were cut in the RD as seen in Figure 1.

## 3. Results

### 3.1. SEM/EBSD Characterization of Cold-Rolled Zn-Cu Alloys

**Figure 2:**
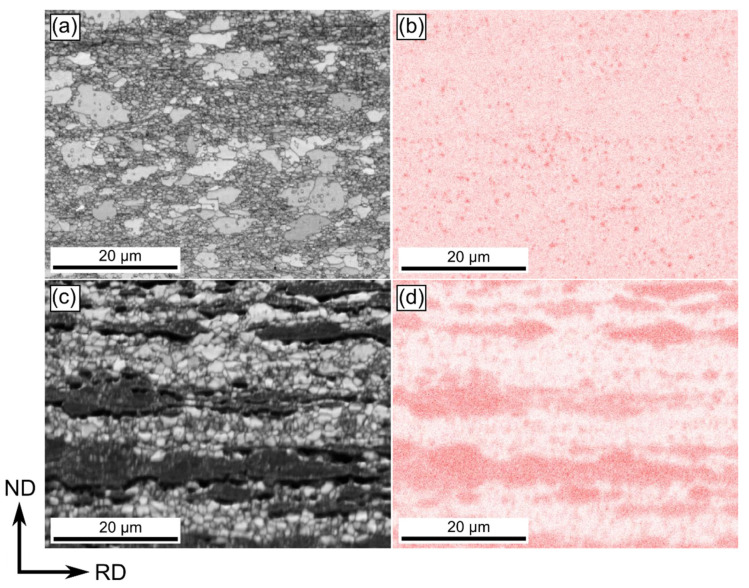
SEM/EBSD image quality map and corresponding Cu distribution map of samples cold-rolled with the reduction rate of 90% Zn-1Cu alloy (**a**,**b**) and Zn-5Cu (**c**,**d**) alloy.

Microstructure of Zn-1Cu alloy deformed by cold-rolling with 90% of reduction was composed of η-Zn grains and small, round-shaped, evenly distributed precipitates rich in Cu. Based on the element distribution map gathered during the EBSD data collection, it was also observed that Cu was present in the solution as well (Figure 2). An increase in Cu addition up to 5 wt.% resulted in a higher volume fraction of the second phase forming almost band-like microstructure elongated in the RD. In Zn-5Cu alloy, except for the larger elongated precipitates, small round ones (as in the Zn-1Cu alloy) were also observed. On the contrary to the Zn-1Cu alloy, depletion of Cu contained in the solution was noticed.

**Figure 3:**
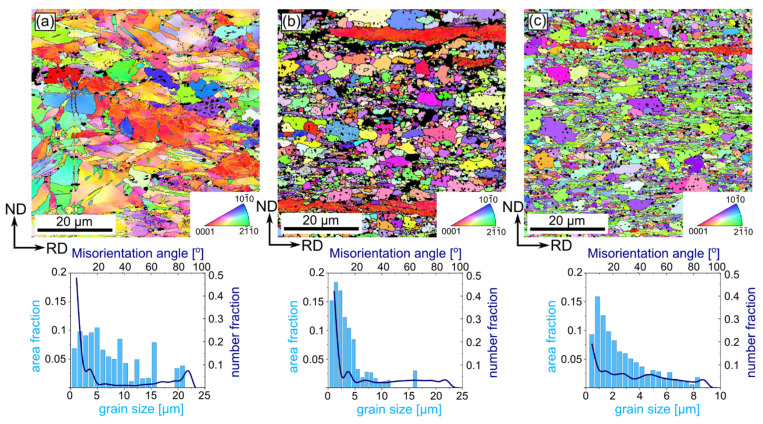
Orientation maps with the corresponding distribution of grain size and misorientation angle of the cold-rolled Zn-1Cu alloy with reduction rate 50% (**a**), 75% (**b**) and 90% (**c**). Dark areas correspond to the second phase and were excluded from the analysis. HAGBs and LAGBs were marked with black and yellow colors, respectively. Orientation maps are color-coded based on the IPF triangle depicted in the right corner with respect to the RD.

**Figure 4:**
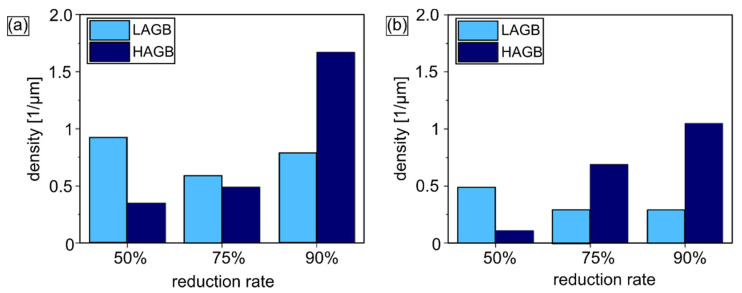
Density of LAGBs and HAGBs obtained for Zn-1Cu (**a**) and Zn-5Cu (**b**) alloys cold-rolled with different reduction rates.

Systematic analysis by means of orientation mapping during multi-pass cold rolling up to 90% of reduction performed for both Zn-1Cu and Zn-5Cu alloys on the ND-RD was carried out in the way to allow the microstructural evolution to be monitored. Thus, additional observation on the particular steps of the cold-rolling process, namely with the reduction rates of 50% and 75%, were chosen. The microstructure of the Zn-1Cu alloy cold-rolled with the reduction rate of 50% was composed of large, slightly elongated in the RD, and η-Zn grains with an average diameter size of 7.7 ± 6 µm. In the orientation map presented in Figure 3a, some twins were also distinguished. Moreover, small secondary phases, seen as black areas on the orientation maps (excluded from the calculations due to the low confidence index value), were observed mostly within grains. Cold rolling of the Zn-1Cu alloy with 50% of the reduction rate caused an accumulation of large number of defects as a higher density of Low Angle Grain Boundaries (LAGBs) compared to High Angle Grain Boundaries (HAGBs) was noticed. An increasing strain provoked a gradual grain refinement as the average values of grain size equaled to 3.6 ± 3 µm and 2.6 ± 2 µm were achieved for 75% and 90% of the reduction rate, respectively. Moreover, higher reduction rates caused the formation of heterogeneous microstructures, composed of grains elongated in the RD, and coarse and ultra-fine grains. The higher the reduction rate was, the greater the heterogeneity that was obtained. Within the elongated grains, a high density of LAGBs was also observed. In most cases those elongated grains possessed the privileged orientation i.e., the <0001> direction aligned along the RD. A constant increase in HAGB density was observed with the increasing reduction rate. It was also observed that, firstly, a density of LAGBs decreased then increased again for the reduction of 90%, which is depicted in Figure 4.

**Figure 5:**
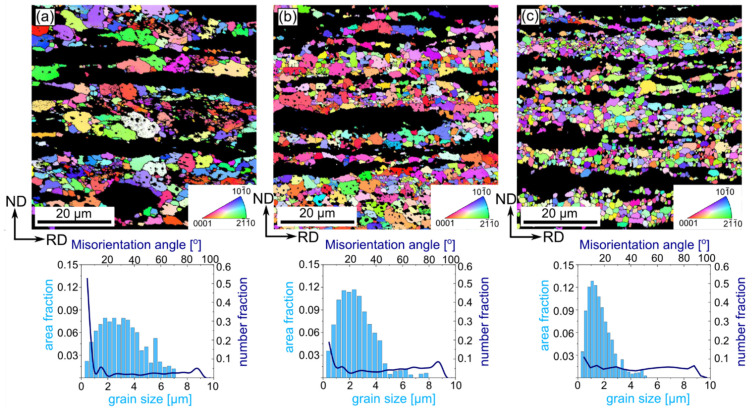
Orientation maps with the corresponding distribution of grain size and misorientation angle of cold-rolled Zn-5Cu alloy with the reduction rate of (**a**) 50%, (**b**) 75% and (**c**) 90%. Dark areas related to the second phase and were excluded form from the analysis. HAGBs and LAGBs were marked with black and yellow colors, respectively. The orientation maps are color-coded based on the IPF triangle depicted in the right corner with the respect to the RD.

An increase of the Cu addition up to 5 wt.% provoked the formation of the intermetallic phase, apart from forming small precipitates, transformed into large, elongated in the RD band-like structure, covering more than 50% of the orientation maps area, depicted in Figure 5. The increased fraction of the second phase contributed to smaller average grain size obtained in the Zn-5Cu alloy compared to the Zn-1Cu alloy. Moreover, the Zn-5Cu alloy possessed more homogeneous distribution of grain size and grain shape, indicating that nearly equiaxed grains were observed after different reductions. While investigating the microstructure evolution of the Zn-5Cu alloy, similar conclusions as in the case of the Zn-1Cu alloy can be drawn. An increasing reduction rate caused gradual grain refinement of Zn alloy with a higher Cu amount. The average grain size of 3.1 ± 2 µm, 2.5 ± 1 µm and 1.6 ± 1 µm were achieved for the reduction rates of 50%, 75% and 90%, respectively. However, in the case of the Zn-5Cu alloy those values are smaller than in the Zn-1Cu alloy. Moreover, the higher the reduction rate, the narrower the range of grain size distribution and the higher the fraction of equiaxed grains. Another similarity elicited by cold rolling was that 50% reduction of the Zn-5Cu alloy resulted in an accumulation of a large density of LAGBs (Figure 4b). An increasing reduction rate decreased the density of LAGBs. Moreover, the number of LAGBs was lower for the Zn-5Cu than for the Zn-1Cu. Furthermore, a smaller density of HAGBs was observed for the alloy with higher Cu addition, what seems unlikely since it is characterized by the smaller grain size. A feasible explanation for this finding can be the fact that the Zn-1Cu alloy wider spread of grain size distribution and numerous elongated, thin grains are observed what increases the overall grain density as compared to the Zn-5Cu alloy. The Zn-1Cu alloy exhibited a high grain boundary density, both LAGB and HAGB, while in the Zn-5Cu alloy, a significant predominance of the HAGB share over LAGB was observed for higher reduction rates (Figure 4).

**Figure 6:**
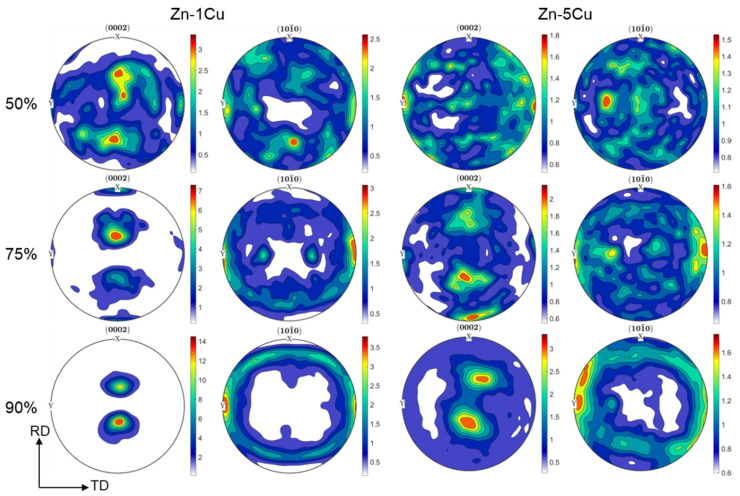
(0002) and (10$\bar{1}$0) pole figures of the Zn-1Cu (left side) and Zn-5Cu (right side) alloys after cold rolling with reduction rate 50% (top row), 75% (middle row) and 90% (bottom row).

Based on the EBSD data, crystallographic texture analysis was also conducted. Figure 6 shows (0002) and (10$\bar{1}$0) pole figures for all the investigated materials. It can be seen that for both Zn-Cu alloys texture gradually evolved with an increasing reduction rate into typical texture observed for cold-rolled hexagonal materials with the c/a ratio higher than the ideal value of 1.633 [18]. In this case, <0001> direction is tilted away from ND against RD about 15–20°. Before such texture was achieved, at the beginning of the cold rolling with 50% of reduction, it was more blurred, but tended to the typical texture for the Zn-1Cu alloy and was more random for Zn-5Cu alloy. The increasing reduction rate to 75% caused another change in texture formation. In the case of the Zn-1Cu alloy, sharper basal pole tilted toward the RD, as well as the additional components at 90° in the RD and the transverse direction (TD), were observed. Similar results were achieved for the Zn-5Cu alloy; however, texture components had lower intensity than for Zn-1Cu alloy. Finally, the typical cold-rolled texture was found for both alloys, except that the Zn-1Cu alloy exhibited higher texture intensity. The received texture implies that plastic deformation of cold rolled Zn-Cu alloys resulted from a combination of basal slip and twinning [18]. 

Another microstructural feature that can be calculated based on SEM/EBSD measurements is the GOS parameter. Usually, a value of about 2° is considered to be specific for recrystallized grains [19]. Recently, Hadadzadeh et al. proposed a new GOS approach to analyze the Dynamic Recrystallization (DRX) and set 5° as a limit value to separate deformed grains from recrystallized ones [19]. Such an approach worked well in our studies and, thus, 5° was used to distinguish DRXed from deformed grains in the cold-rolled Zn-Cu alloys. Moreover, for all materials the same scale was fixed with maximum value of 25 marked with red color indicating the highest heterogeneity within the particular grain. 

**Figure 7:**
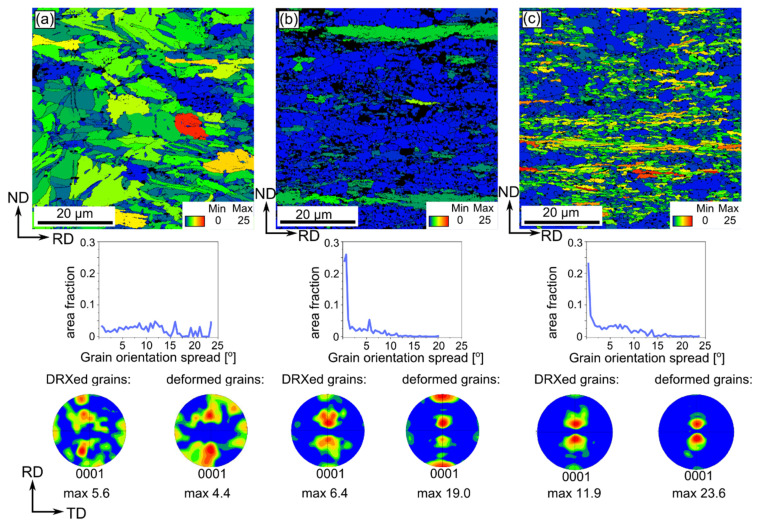
GOS maps with the parameter distribution and below (0001) pole figures of separate based on GOS parameter fraction of DRXed grains (GOS ≤ 5°) and deformed grains (GOS > 5°) of the cold-rolled Zn-1Cu alloy with the reduction rates of 50% (**a**), 75% (**b**) and 90% (**c**).

**Figure 8:**
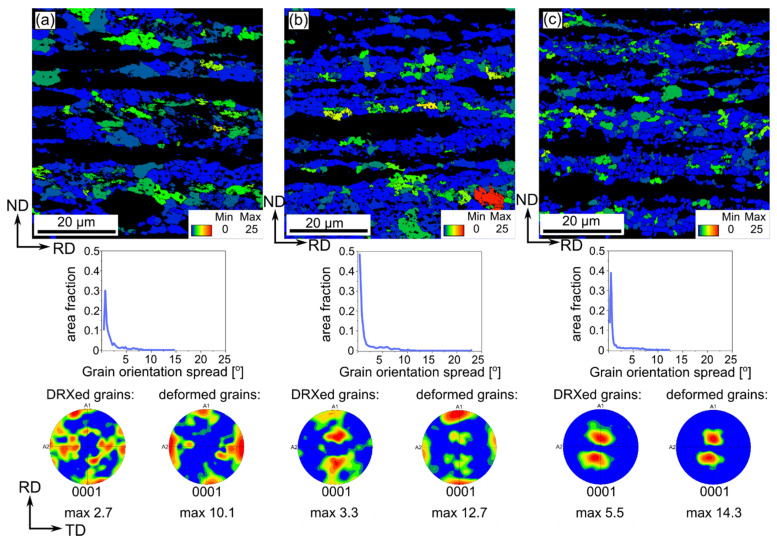
GOS maps with the parameter distribution and below (0001) pole figures of separate based on GOS parameter fraction of DRXed grains (GOS ≤ 5°) and deformed grains (GOS > 5°) of the cold-rolled Zn-5Cu alloy with the reduction rates of 50% (**a**), 75% (**b**) and 90% (**c**).

GOS maps, depicted in Figure 7 and Figure 8, indicate that the Zn-Cu alloys cold-rolled with the reduction of 50% exhibited the highest GOS parameter. The increasing reduction rate caused that the number of recrystallized grains increased. A higher GOS parameter was observed for the Zn-1Cu alloy as compared to the Zn-5Cu alloy, regardless of the reduction rate. Moreover, the GOS maps indicated that larger distortion was present within elongated grains, but they are not the only exception. Some parts of coarse grains also exhibited local misorientation. This may also be seen on the GOS charts, indicating that with the increasing reduction rate, the area fraction of grains with the GOS value 2–5° increased, which also corresponds to the DRXed deformed grains [19].

After dividing grains into two groups, the crystallographic microtexture was determined. The results revealed that DRXed grains followed the texture of deformed grains, even though the texture was more blurred and of lower intensity.

Table 1 summarizes the microstructural characteristics of cold-rolled Zn-Cu alloys obtained based on EBSD measurements. 

### 3.2. TEM Nanoscale Characterization of Cold-Rolled Zn-Cu Alloys

**Figure 9:**
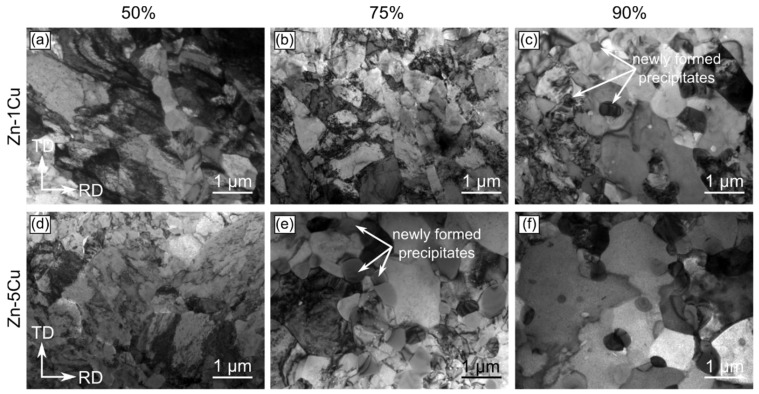
TEM/BF microstructure images of Zn-1Cu and Zn-5Cu alloys subjected to cold-rolling with reduction rate of 50% (**a**,**d**), 75% (**b**,**e**) and 90% (**c**,**f**).

**Figure 10:**
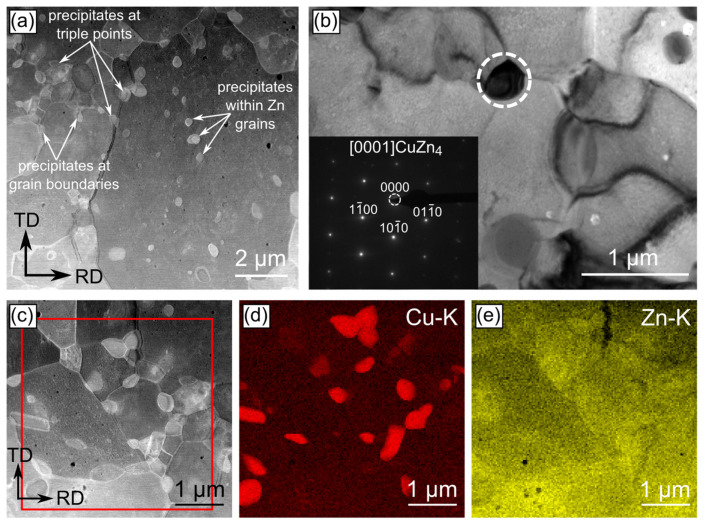
STEM/HAADF (**a**,**c**) and TEM/BF (**b**) microstructure images presenting the CuZn_4_ precipitates within the Zn-1Cu alloy subjected to cold-rolling with reduction rate of 90% as well as the EDS maps showing distribution of Cu (**d**) and Zn (**e**).

TEM investigations were performed in order to provide complementary information to the data obtained during SEM/EBSD experiments. Simultaneously, they were focused on the nanoscale characterization of the areas excluded from the orientation maps. The observations executed in the bright-field mode indicated that the microstructure of both the Zn-1Cu and Zn-5Cu alloys cold-rolled with the reduction rate of 50% is composed of large η-Zn grains with uniformly distributed crystal defects of high density (Figure 9a,d). With the increase in the deformation rate, the microstructure remodeling may be noticed as the defects tended to rearrange and group into the low angle grain boundaries (Figure 9b,e). Cold rolling with the reduction rate of 90% resulted in the formation of the microstructure with greater number of defect-free crystallites as compared to lower reduction rates (Figure 9c,f). For the reduction rates of 90% and 75% for the Zn-1Cu and Zn-5Cu alloys, respectively, one may also observe the formation of the small precipitates, of equiaxed or lenticular shape, with a size approaching 1 µm. Simultaneously, the number of these precipitates increased with an increase in the cold-rolling reduction rate. The STEM/HAADF imaging revealed that their formation occurred not only at grain boundaries and triple points, but also within the η-Zn crystallites, thanks to the contrast, originating from different atomic numbers of η-Zn and the precipitates (Figure 9a). The phase analysis of small precipitates was done through the indexing of the spots presented in the SAED pattern, recorded from one of those crystallites, and allowed to identify them as the ε-CuZn_4_ phase (Figure 10b) [20]. In addition, the EDS characterization aimed at determining the chemical elements distribution and the quantitative analysis of precipitates was also carried out by acquiring the spectra from different measurement points (Figure 10). The EDS mapping confirmed the maxima of Cu concentration within the CuZn_4_ precipitates pinpointed by the SAED phase analysis. The quantitative chemical analysis enabled for a determination of their chemical composition as 12.3 ± 1.3% and 87.7 ± 1.3% (in at.%) for Cu and Zn, respectively. Moreover, a small amount of Cu was also dissolved in the η-Zn grains, forming a solid solution.

### 3.3. Mechanical Properties of Cold-Rolled Zn-Cu Alloys

Mechanical tests revealed that the highest strength was observed for the Zn-5Cu alloy cold-rolled with 50% of reduction i.e., YS = 156 MPa and UTS = 240 MPa. In the Zn-1Cu alloy, the highest YS = 142 MPa and UTS = 203 MPa were also achieved after cold-rolling with the reduction rate of 50%. Further increase in the reduction rate caused a decrease in mechanical strength for both materials, yielding UTS = 143 MPa and 116 MPa for the Zn-1Cu alloy after cold rolling with the reduction of 75% and 90%, respectively, and for the Zn-5Cu alloy: UTS = 182 MPa (reduction rate 75%) and UTS = 144 MPa (reduction rate 90%). A higher strength obtained for the Zn-5Cu alloy can be substantiated by the higher amount of Cu addition, which contributed to the formation of numerous second phases, strengthening the material. On the contrary, the increasing reduction rate resulted in a substantial increase in elongation of the Zn-Cu alloys and in the case of Zn-1Cu cold-rolled with 90% of reduction, the superplastic effect was observed (E = 272%).

**Figure 11:**
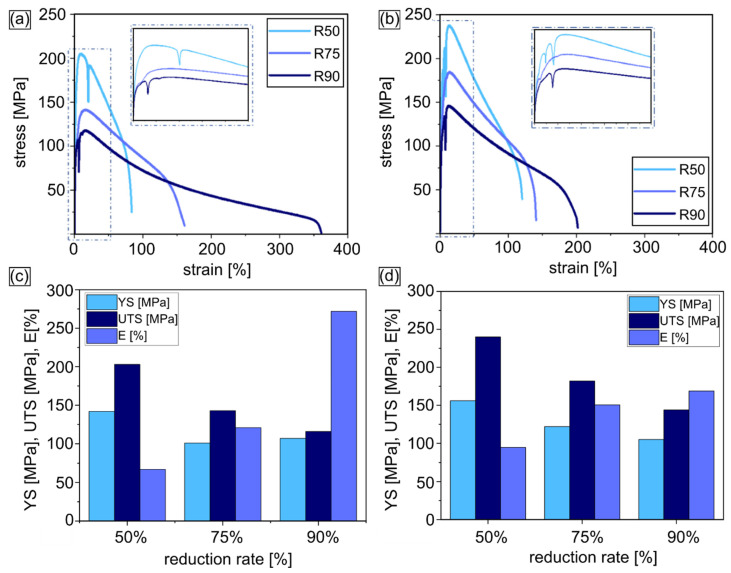
Strain-stress curves (for better visibility dashed line area was magnified and showed above original curves) of cold-rolled with different reduction rate Zn-1Cu alloy (**a**) and Zn-5Cu alloy (**b**) and corresponding mechanical properties (**c**,**d**).

Stress-strain curves showed that the Zn-5Cu alloy exhibited a wide range of strain-hardening to about 15%, which was higher than for the Zn-1Cu alloy of at least 5% (Figure 11). After reaching a critical value, the strain-softening effect was observed for all of the investigated materials. Moreover, single or multiple serrations were observed on strain-stress curves for the reduction rates of 50% and 90%, which are assumed to be related to twinning. 

## 4. Discussion

### 4.1. Continuous Dynamic Recrystallization in Cold-Rolled Zn-Cu Alloys

Investigations of microstructure evolution of the Zn-Cu alloys during cold rolling up to 90% of reduction rate were performed in the present work. Plastic deformation contributed to the significant accumulation of structural defects, especially at the very beginning. It has been proved by the high values of the GOS parameter as well as the high density of LAGBs for the Zn-Cu alloys with a reduction of 50%. With the increasing reduction rate, gradual transformation of LAGBs into HAGBs was observed, as revealed by the EBSD and TEM studies, manifesting the occurrence of continuous dynamic recrystallization process (CDRX) [21]. Such mechanism allowed for grain refinement of the Zn-Cu alloys, where small equiaxed and defect-free grains were seen in both the orientation maps and TEM microstructure images. Similar microstructural evolution, including the reduction of the LAGBs density in favor of a higher amount of small dynamically recrystallized (DRXed) grains and higher HAGBs density, was shown for the cold-rolled Zn-Ag-Mg alloy for progressively increased cold rolling reduction rates resulting ultimately in superior plasticity after the reduction rate of 90% [22]. Additionally, due to the investigation of the GOS parameter and separation of a fraction of DRXed grains from deformed ones, it was also possible to support the evidence of CDRX, since the obtained texture of the DRXed grains followed the texture of deformed grains. A more pronounced effect of CDRX was observed for the Zn-5Cu alloy, where systematic drop in LAGBs density in addition to the decrease of the grain size with increasing reduction rate was reported. In the case of Zn-1Cu, the effect of CDRX in forming refined, equiaxed micro-structure was limited due to the static recrystallization process, which caused grain growth and heterogeneous microstructure. The contribution of CDRX to grain refinement of Zn-based materials was also observed for other methods of plastic deformation such as Equal Channel Angular Pressing (ECAP) or hydrostatic extrusion [9,23,24,25,26,27].

### 4.2. Superior Plasticity of Cold-Rolled Zn-Cu Alloys

It is suggested that DRX not only determined the microstructural changes but also affected mechanical properties. The observed decrease in strength of Zn-Cu alloys with the increasing reduction rate was caused by DRX, which is known to play a crucial role in softening during deformation [21,28]. Simultaneously, DRX largely contributes to better formability of a material [29]. The results revealed that with the increasing reduction rate, a remarkable increase in elongation of the Zn-Cu alloys was achieved. Similar observations were reported by Liu et al. while subjecting pure Zn to compression at ambient temperature [29,30]. They compressed as-cast pure Zn to the true strain of 161% and investigated microstructure on different stages of deformation. Their observation revealed that such outstanding plasticity originated from the abundant operation of CDRX supported by twinning-induced dynamic recrystallization (TDRX), which leads to the relaxation of local stresses [29]. In our work, the predominant effect of CDRX was reported, however TDRX seems to be active as well, since on the stress-strain curves the indication of twins was observed. 

Thus, it is evident that the occurrence of CDRX plays a significant role in materials’ formability. However, the results indicate that the effect of DRX on superior plasticity can be intensified by the static recrystallization process. Increasing strain rate caused generation of heat, which for Zn—a material with recrystallization temperature close to room temperature—was high enough to start the recrystallization process of some grains after a single pass of cold-rolling. Thus, this is the reason for obtaining the heterogeneous microstructure of the Zn-1Cu alloy deformed with the reduction rate of 75% and 90%. Increasing amount of Cu addition up to 5 wt.% suppressed the described effect, due to the presence of CuZn_4_ phases, forming the band-like structures, which were a barrier for dislocation movement and prevented grain growth. It was found that CuZn_4_ phase can serve as a Particle Stimulated Nucleation place and, therefore, promotes the recrystallization process [15]. However, our results indicate that the bands of the CuZn_4_ phase also act as obstacles for grain boundary movement and inhibit grain growth. 

Due to the increased number of ε-phase precipitates forming a band-like microstructure, the Zn-5Cu alloy exhibited a more homogeneous distribution of grain size and grain shape than the Zn-1Cu alloy. The second phase contributed to the higher strength of the Zn-5Cu alloy and greater strain-hardening. Despite strengthening by second phases the DRX occurred, leading to stress relaxation and provoked materials softening. The lower value of elongation in the Zn-5Cu alloy, beside hindering dislocation motion due to the ε-phase, can be explained by smaller grain size and, as a consequence, the increased fraction of HAGB, another obstacle for dislocation movement. 

**Figure 12:**
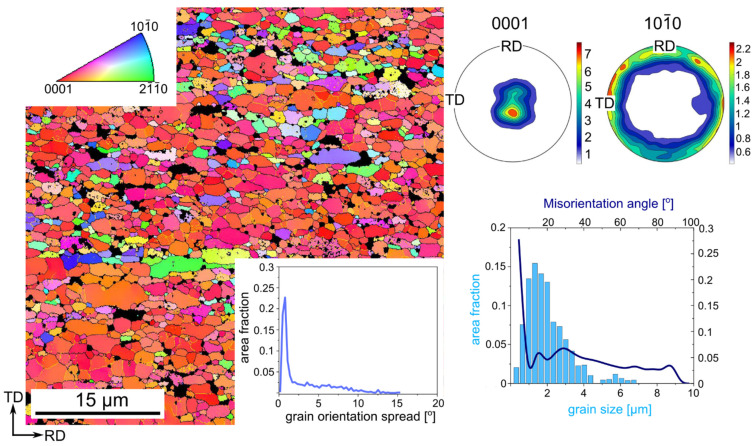
Orientation map with the GOS chart, (0001) and (10$\bar{1}$0) pole figures and distribution of grain size and misorientation angle of the cold-rolled Zn-1Cu alloy with the reduction rate of 90% acquired from the necking of the sample after tensile test. Dark areas related to the CuZn_4_ phase were excluded from the analysis. HAGBs and LAGBs were marked with black and yellow colors, respectively. The orientation map is color-coded based on the IPF triangle depicted in the right upper corner with respect to the ND.

As a matter of fact, the coarse grains present in the Zn-1Cu alloy seem to be beneficial for plasticity improvement. The coarse-grain twinning is more likely to occur, which subsequently can enhance the effect of DRX, resulting ultimately in a higher elongation. Additionally, larger grain size increases a mean free path for dislocation movement so the dislocation can form sub-grains divided by LAGBs and gradually transform these LAGBs into HAGBs. Thus, it is possible for large grains to get refined through CDRX and some newly DRXed grains can grow in size and subsequently undergo deformation once more and eventually recrystallize statically. Therefore, this process can be repeated and leads to an improvement in the Zn-Cu formability. This hypothesis can be supported by comparing microstructure of the Zn-1Cu alloy cold rolled with a reduction rate of 75% and that of 90%. In both cases the heterogeneous microstructure was observed and for the reduction of 90%, the number of LAGBs increased as well as the number of refined grains, as compared to the reduction of 75%. In order to find a possible explanation for what may have stopped this process, the additional observation of the Zn-1Cu alloy with 90% of reduction after the uniaxial tensile test was performed. The orientation map presented in Figure 12 showed the microstructure with even more refined grains than in the sample before mechanical testing as the average grain size 2 ± 1 µm, was achieved. Moreover, the obtained grains possessed a strong basal texture, in which the basal planes are parallel to the rolling plane. It was reported by Liu et al. that such texture is a “hard-orientation” and thus enables basal slip and twinning to operate [29]. Furthermore, it was shown that the basal texture retards DRX. Our investigation can support such an observation, since, on the orientation maps obtained for the Zn-1Cu alloy cold-rolled with the 75% of reduction rate, elongated grains also possessed hard orientation and were still present on the orientation maps with the reduction of 90%, but were finer. Thus, during tensile tests, due to initial, typical cold rolling texture, the dislocation slip operated in the basal system, leading eventually to the sharp basal texture. At the beginning, the dislocations movement provoked hardening and subsequently, due to the recovery process, caused their annihilation and formation of sub-grains. Under the strain, sub-grains can rotate into HAGBs and CDRX operates, which can explain the observed grain refinement after tensile testing. When finally hard orientation is achieved, there is no possible stress relaxation by DRX and, thus, necking appears and the sample fractures. This texture rotation can be supported by mechanical twinning as its presence was confirmed on the stress-strain curves, which could also facilitate DRX [29,30]. In general, it is believed that the observed superior plasticity in the Zn-1Cu alloy cold-rolled with the reduction of 90% was provoked by dynamic recovery and recrystallization processes, which eliminates the effects of hardening elicited by deformation. The effects of hardening and softening compete with each other until the hard orientation occurs and hinders both of the effects.

**Figure 13:**
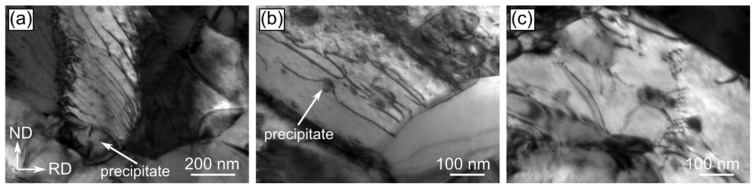
TEM/BF microstructure of the cold-rolled Zn-1Cu alloy with 90% of reduction after uniaxial tensile test: pile-up of dislocations around the CuZn_4_ precipitate (**a**), bowing of dislocation around the CuZn_4_ (**b**) and dislocations annihilation into sub-grain around the CuZn_4_ (**c**).

The room-temperature superplastic effect in Zn-Cu alloys was already proven while performing ECAP method or cold rolling [9,31]. Bednarczyk et al. obtained 510% of elongation at strain rate 1 × 10^−4^ s^−1^ in the Zn-0.5Cu alloy [9], meanwhile Mostaed et al. at the same rate observed 470% of elongation at the same rate for the cold-rolled Zn-1Cu alloy. While the effect itself is well established as determined by a series of static tensile tests at different strain rates, the clear source of the mechanisms behind the superplasticity effect observed in Cu-bearing Zn alloys is not been unambiguously concluded. The authors of the abovementioned works were more focused on characterizing the initial microstructure prior to the tensile tests with different strain rates, which was strongly justified since grain size is one of the key factors affecting the superplastic effect [32]. Bednarczyk et al. concluded that superplastic effect was generally caused by grain boundary sliding (GBS). This mechanism was not considered in our investigations due to texture analysis. GBS should cause texture randomization or at least some intensity weakening [33] and, in our studies, with the increasing reduction rate, the texture intensity increased as well. The later work reported by Mostaed et al. provides an explanation that the superplastic effect of the cold-rolled Zn-1Cu alloy is possible due to the phase boundary sliding, and the in-creased volume fraction of the ε-phases resulted in more sites for the activation of Zn/CuZn_4_ boundary sliding rather than Zn/Zn glides, because cold rolling provoked strain-induced precipitations. Our results also proved strain-induced precipitations and with the increasing reduction rate, a pronounced number of new precipitates were observed. However, our other results also revealed that CuZn_4_ precipitates tend to strengthen the material and they are an obstacle to dislocation movement. Firstly, it can be proved by the fact that the Zn-5Cu alloy possessed lower plasticity than the Zn-1Cu alloy. Secondly, a more detailed microstructural investigation in the nanoscale done by TEM observation of the Zn-1Cu alloy after the tensile test revealed numerous interactions of dislocations with the CuZn_4_ phase. The interactions between dislocations and second phases were observed during TEM investigations of the Zn-1Cu alloy cold-rolled with 90% of the reduction after the uniaxial tensile test. The pile-up of dislocations on the ε-phase as well as dislocation bowing around particle were found and presented in Figure 13. However, our uniaxial tensile tests were conducted with only one strain rate, while in [9,31] the authors performed more comprehensive mechanical tests covering the strain rate range of 10^−5^÷10^0^ s^−1^, thus the possibility of other mechanisms controlling superplastic effect cannot be excluded. Nevertheless, our investigation proved the substantial contribution of DRX in achieving remarkable plasticity and thus should not be neglected in future work concerning the superplasticity of Zn-based materials. 

## 5. Conclusions

The presented results revealed the origin for superior plasticity and general improvement in the formability of Zn alloys by studying microstructural evolution. The dynamic recrystallization process can be crucial in microstructural formation and, hence, it governs the mechanical properties. The observed continuous dynamic recrystallization process is not only responsible for grain refinement of Zn-Cu alloys but also significantly contributes to achieving remarkable plasticity and even the superplastic effect for the Zn-1Cu alloy cold-rolled with the reduction of 90%. This was possible due to the heterogeneous microstructure. In coarse grains, dislocation movement is still possible, although due to dynamic recovery, dislocations annihilate and form sub-grains. The sub-grain boundaries eventually rotate into HAGBs due to the CDRX mechanism. Next, some grains can grow again due to static recrystallization and the process in some specific conditions can happen continuously.

## Figures and Tables

**Table 1:** List of microstructural characteristics of cold-rolled Zn-1Cu and Zn-5Cu alloys.

	Zn-1Cu Alloy			Zn-5Cu Alloy		
Reduction rate	50%	75%	90%	50%	75%	90%
Average grain size [µm]	7.7 ± 6	3.6 ± 3	2.6 ± 2	3.1 ± 2	2.5 ± 1	1.6 ± 1
Average misorientation angle [°]	25	27	34	25	43	44
Average GOS [°]	9.9 ± 6	2.8 ± 3	4.8 ± 5	1.9 ± 2	1.8 ± 3	1.7 ± 2
HAGB density [1/µm]	0.35	0.49	1.67	0.11	0.69	1.05
LAGB density [1/µm]	0.93	0.59	0.79	0.49	0.29	0.29
Fraction of CuZn_4_ [%]	6.1	26.6	10.5	60.5	50.7	56.1

## Data Availability

The data presented in this study are available in repository of corresponding author.
